# Real-Time Diabetic Retinopathy Severity Score Level versus Ultra-Widefield Leakage Index-Guided Management of Diabetic Retinopathy: Two-Year Outcomes from the Randomized PRIME Trial

**DOI:** 10.3390/jpm11090885

**Published:** 2021-09-04

**Authors:** Hannah J. Yu, Justis P. Ehlers, Duriye Damla Sevgi, Margaret O’Connell, Jamie L. Reese, Sunil K. Srivastava, Charles C. Wykoff

**Affiliations:** 1 Retina Consultants of Texas, Retina Consultants of America, Houston, TX 77030, USA; hannahyu2019@gmail.com; 2 Tony and Leona Campane Center for Excellence in Image-Guided Surgery and Advanced Imaging Research, Cole Eye Institute, Cleveland Clinic, 9500 Euclid Ave/i32, Cleveland, OH 44195, USA; sevgid@ccf.org (D.D.S.); oconnem6@ccf.org (M.O.); reesej3@ccf.org (J.L.R.); SRIVASS2@ccf.org (S.K.S.); 3 Blanton Eye Institute & Weill Cornell Medical College, Houston Methodist Hospital, 6560 Fannin St., Suite 750, Houston, TX 77030, USA

**Keywords:** diabetic retinopathy, anti-vascular endothelial growth factor, diabetic retinopathy severity scale, panretinal leakage index

## Abstract

The prospective PRIME trial applied real-time, objective imaging biomarkers to determine individualized retreatment needs with intravitreal aflibercept injections (IAI) among eyes with diabetic retinopathy (DR). 40 eyes with nonproliferative or proliferative DR without diabetic macular edema received monthly IAI until a DR severity scale (DRSS) level improvement of ≥2 steps was achieved. Eyes were randomized 1:1 to DRSS- or PLI- guided management. At the final 2-year visit, DRSS level was stable or improved compared to baseline in all eyes, and mean PLI decreased by 11% (*p* = 0.73) and 23.6% (*p* = 0.25) in the DRSS- and PLI-guided arms. In both arms, the percent of *pro re nata* (PRN) visits requiring IAI was significantly higher in year 2 versus 1 (*p* < 0.0001). The percent of PRN visits receiving IAI during year 1 was significantly correlated with the percent of PRN visits with IAI during year 2 (*p* < 0.0001). Through week 104, 77.4% of instances of DRSS level worsening in the DRSS-guided arm were preceded by or occurred alongside an increase of PLI. Overall, consistent IAI re-treatment interval requirements were observed longitudinally among individual patients. Additionally, PLI increases appeared to precede DRSS level worsening, highlighting PLI as a valuable biomarker in the management of DR.

## 1. Introduction

Diabetic retinopathy (DR) is a leading cause of preventable vision loss [1]. In the setting of diabetic macular edema (DME) with visual loss, multiple studies have demonstrated the remarkable visual and anatomic value of consistent anti-vascular endothelial growth factor (VEGF) pharmacotherapy initiated early in the disease process [2,3,4,5]. Similarly, prospective studies involving eyes with DR without DME, both proliferative DR (PDR) [6,7,8,9] and nonproliferative DR (NPDR) [10,11], have repeatedly demonstrated clinically meaningful anatomic benefits with repeated anti-VEGF treatment compared to laser or observation. While fixed interval anti-VEGF dosing among a population has frequently been employed in clinical trials [2,3,10], this is rarely applied to routine clinical practice. Rather, specific examination and/or imaging-based biomarkers of disease activity are typically applied to guide dosing frequency using an individualized, patient-centric approach.

In the context of DME, the objective endpoint of anti-VEGF therapy is typically fluid resolution on optical coherence tomography (OCT), despite the imperfect correlation between visual acuity and OCT-based fluid status [12,13]. In comparison, OCT-based imaging is typically of limited utility in the management of eyes with DR without DME, because by definition they have no, or limited, central intraretinal or subretinal fluid. There is a need for objective, quantifiable and validated biomarkers of DR disease activity that can be utilized in clinical practice to guide dosing intervals among patients with DR being managed with anti-VEGF pharmacotherapy.

The Intravitreal Aflibercept as Indicated by Real-Time Objective Imaging to Achieve Diabetic Retinopathy Improvement (PRIME) trial was designed to evaluate the utilization of real-time assessments of DR severity levels on fundus photography and panretinal leakage index (PLI) on wide-field fluorescein angiography in guiding anti-VEGF retreatment decisions in the management of DR.

In the first year of the study, while no meaningful differences in visual or anatomic outcomes were observed between the two re-treatment strategies [14], results suggested that PLI worsening may precede DR severity worsening. In year 2 of the PRIME trial, re-treatment strategies remained consistent, but assessments were performed every other month instead of monthly. The purpose of the current work is to describe outcomes from the 2nd and final year of the PRIME trial.

## 2. Methods

The PRIME trial was a randomized, phase 2 clinical trial (ClinicalTrials.gov, NCT03531294). Institutional review board (IRB; Advarra IRB, Columbia, MA, USA) and ethics committee approval was obtained for this Health Insurance Portability and Accountability Act-compliant trial adhering to the tenets of the Declaration of Helsinki. Data were collected at the Retina Consultants of Texas (Houston and The Woodlands, TX, USA). All participants provided written informed consent prior to enrollment. Study methods were previously reported [14]. Briefly, 40 subjects with treatment-naïve diabetic retinopathy (DR) with a DR severity scale (DRSS) level of 47A to 71A as determined by the central reading center (CRC; Cole Eye Institute, Cleveland Clinic, Cleveland, OH, USA) were randomized 1:1 to a DRSS-guided or PLI-guided arm. Enrollment of eyes with PDR was limited to 50% of the total population. Subjects were excluded if they had spectral domain-OCT central subfield thickness (CST) of more than 320 µm in the study eye or had central DME causing loss of visual acuity (VA).

Subjects were assessed monthly (28 ± 7 days) through week 52; real-time CRC grading of DRSS and PLI was performed using Early Treatment Diabetic Retinopathy Study (ETDRS) 7-standard-field fundus photography imaging (FF4 fundus camera; Zeiss Meditec, Dublin, CA, USA) and ultra-widefield fluorescein angiography (UWFA; 200Tx device; Optos, Dunfermline, United Kingdom). The ETDRS scale [15] was used to grade DRSS (35 = mild NPDR; 43 = moderate NPDR; 47 = moderately severe NPDR; 53 = severe NPDR; 61 = mild PDR; 65 = moderate PDR; 71 and 75 = high-risk PDR; 81 and 85 = advanced PDR). All study eyes received monthly 2.0 mg intravitreal aflibercept injection (IAI) until a DRSS level improvement of ≥2 steps relative to baseline was achieved. PLI at the visit at which a DRSS level improvement of ≥2 steps was achieved was defined as the threshold leakage. For subjects randomized to the DRSS-guided arm, monthly re-treatment with IAI was initiated if a 1-step worsening of DRSS occurred compared to the best DRSS level achieved; treatment was stopped when the best or better DRSS level was again achieved. For subjects randomized to the PLI-guided arm, monthly re-treatment with IAI was initiated if PLI increased to 50% or higher of the difference between the baseline PLI and threshold PLI. Treatment was stopped when PLI decreased to the threshold value, or less. The PLI-guided protocol was optimized with multiple iterations during the early stage of the study, and the majority of assessments were performed using the protocol described above. Beginning at week 52, the interval between visits was lengthened to every other month (56 ± 14 days) and continued through the final visit at week 104, during which need for IAI re-treatment continued to be assessed using the pre-specified DRSS and PLI guidelines for each randomized arm.

*Pro re nata* (PRN, as needed) visits were defined as visits occurring after the initial ≥2 step DRSS improvement was made. PRN IAI were defined as IAI occurring at PRN visits.

The primary outcome measure of the PRIME trial was the incidence of DR-related adverse events among DR subjects receiving IAI. Secondary outcome measures included changes in DRSS level and PLI, number of IAI received, and changes in visual and anatomic measures. Observed data are reported; for subjects who did not complete the week 104 endpoint, corresponding data are included until withdrawal. Statistical analysis was performed using R version 3.5.2 (R Foundation, Vienna, Austria). One-sample and 2-sample paired and unpaired Student’s *t*-tests were used to compare continuous variables, and Fisher’s exact test was used to compare proportions. Pearson’s correlation was used to assess linear relationships between variables.

## 3. Results

40 eyes from 40 subjects were enrolled and randomized in PRIME with a DRSS level of 47 to 71. As reported, baseline demographics appeared well-balanced between the DRSS- and PLI-guided arms [14]. 29 eyes (DRSS-guided = 13; PLI-guided = 16) entered into year 2 of the trial, and 25 eyes (DRSS-guided = 12; PLI-guided = 13), representing 62.5% of enrolled subjects, completed the week 104 visit. During year 2 of PRIME, 2 (6.9%) subjects died, 1 (3.4%) withdrew due to concerns regarding coronavirus disease 2019 (COVID-19), and 1 (3.4%) was loss to follow up (LTFU). The following data focuses on eyes that entered into year 2 of the PRIME trial. Among the 203 possible visits in year 2 for the 29 subjects who entered year 2, 163 (80.3%) were completed.

### 3.1. Re-Treatment Requirements in Year 2

**Figure 1:**
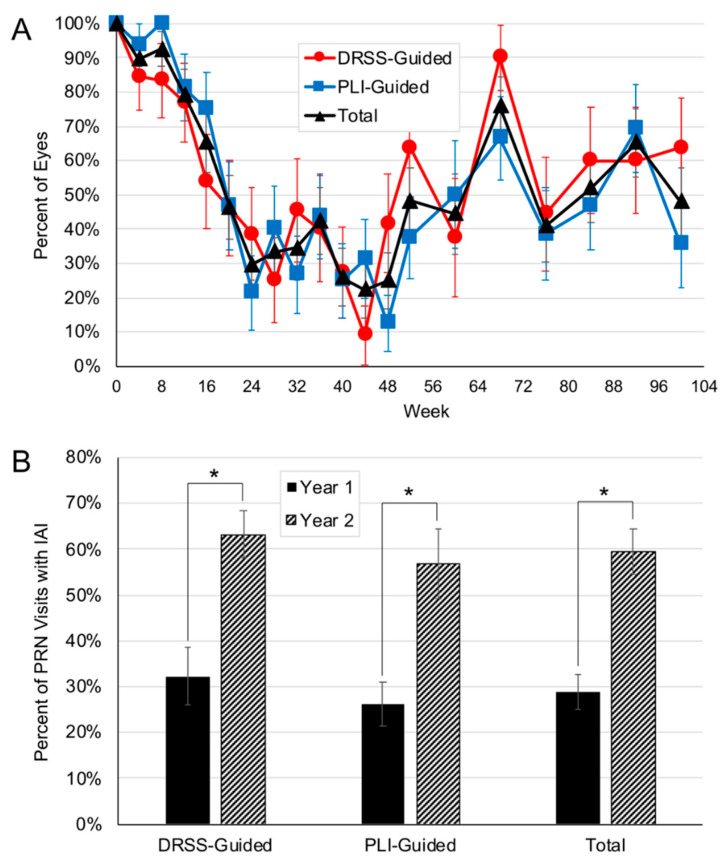
Intravitreal aflibercept injections (IAI) administered through week 104. (**A**) Percent of eyes treated at each visit in the diabetic retinopathy severity scale (DRSS)-guided and the panretinal leakage index (PLI)-guided arms. (**B**) Percent of *pro re nata* (PRN) visits with IAI administration in year 1 and year 2. In the DRSS-guided arm, 32.2% and 63.2% of PRN visits resulted in IAI administration in year 1 and year 2, respectively. In the PRN-guided arm, 26.1% and 56.8% of PRN visits resulted in IAI administration in year 1 and year 2, respectively. Asterisks indicate statistically significant differences between groups.

Cumulatively through week 104, the DRSS-guided arm received a mean total 9.7 IAI (range, 6 to 17) and the PLI-guided arm received 9.8 IAI (range, 6 to 16; *p* = 0.95; Figure 1A). From week 52 through week 104, eyes received a mean 3.3 (range, 1 to 6) and 2.9 (range, 0 to 7) IAI (*p* = 0.62) in the DRSS-guided and PLI-guided arms respectively. In the DRSS-guided arm, 32.2% and 63.2% of PRN visits resulted in IAI re-treatment in year 1 and year 2, respectively (*p* < 0.0001); in the PLI-guided arm, 26.1% and 56.8% of PRN visits resulted in IAI re-treatment in year 1 and year 2 (*p* < 0.0001; Figure 1B). No significant differences were observed between the two arms related to the proportion of PRN visits resulting in IAI re-treatment in year 1 (*p* = 0.45) or year 2 (*p* = 0.5). Overall, after achieving the pre-defined threshold of ≥2-step DRSS level improvement with initial monthly dosing, a mean 5.9 (range, 3 to 11) and 5.4 (range, 3 to 11) PRN IAI were administered to the DRSS- and PLI-guided arms, respectively (*p* = 0.65), over a mean 13.8 ± 2.9 and 13.8 ± 2.7 PRN visits; yielding an average IAI re-treatment interval of every 3.5 months (range, 1.1 to 14). Consistent with this, eyes in the DRSS- and PLI-guided arms had a mean 4.5 (range, 2 to 8) and 4.4 (range, 2 to 12) months between their last monthly IAI and their first PRN IAI (*p* = 0.93); eyes with baseline NPDR and PDR had a mean 4.9 (range, 2 to 12) and 4.1 (range, 2 to 8) months between their last monthly IAI and their first PRN IAI (*p* = 0.41).

**Figure 2:**
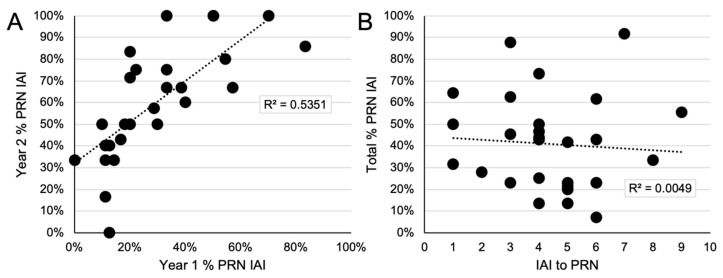
Linear correlations between intravitreal aflibercept injection (IAI) administrations. (**A**) The percent of *pro re nata* (PRN) visits resulting in IAI during year 1 versus the percent of PRN visits resulting in IAI during year 2. A significantly positive linear correlation was observed (R = 0.73, *p* < 0.0001; black dotted line). (**B**) The percent of IAI required to achieve a ≥2-step DRSS improvement versus the total percent of PRN visits requiring IAI through year 2. No significant correlation was observed (R = −0.07, *p* = 0.73; black dotted line).

A relationship was identified among patient-level re-treatment requirements in the first and second years. Specifically, the percent of PRN IAI given during year 1 was significantly correlated with the percent of PRN IAI given during year 2 (R = 0.73, *p* < 0.0001; Figure 2A). In comparison, the absolute number of IAI needed to achieve the initial ≥2-step DRSS level improvement was not found to be correlated with the percent of PRN IAI given (R = −0.07, *p* = 0.73; Figure 2B).

### 3.2. DRSS and PLI Outcomes through Year 2

**Figure 3:**
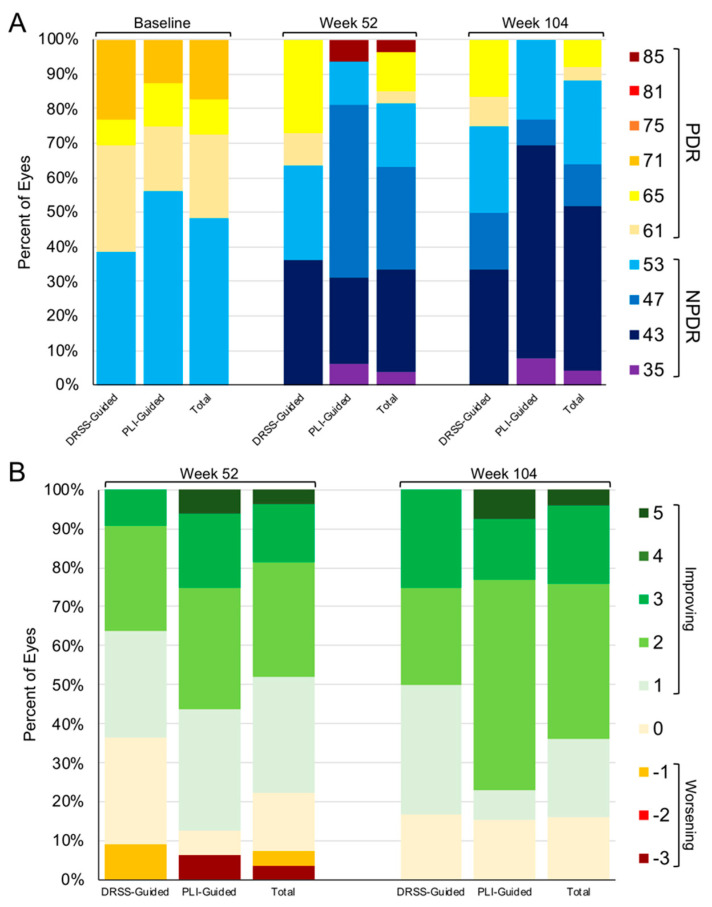
Diabetic retinopathy severity scale (DRSS) levels through week 104. (**A**) Absolute DRSS levels at baseline, week 52, and week 104 in the DRSS-guided and panretinal leakage index (PLI)-guided arms. (**B**) Change in DRSS level compared to baseline at week 52 and week 104 in the DRSS- and PLI-guided arms.

Through week 104, DRSS level stabilized or improved among 16% and 84% of all subjects who completed the week 104 visit (Figure 3) respectively; no subject experienced a worsening of DRSS level at week 104 compared to baseline. 6 (50%) and 10 (76.9%) eyes in the DRSS- and PLI-guided arms experienced a ≥2-step DRSS improvement at week 104 compared to baseline, respectively (*p* = 0.23). Through week 104, one (3.4%) subject never achieved ≥2-step DRSS improvement; this subject missed 12 of 21 visits (57.1%). In the DRSS-guided arm, 8 (61.5%) eyes had PDR at baseline; at week 104, 3 (25%) had PDR, 4 (50%) had NPDR, and 1 (12.5%) was LTFU (NPDR at last observed visit). In the PLI-guided arm, 7 (43.8%) eyes had PDR at baseline; at week 104, 0 had PDR, 4 (57.1%) had NPDR, and 3 (42.9%) were LTFU (2 were NPDR and 1 was PDR at last observed visit).

**Figure 4:**
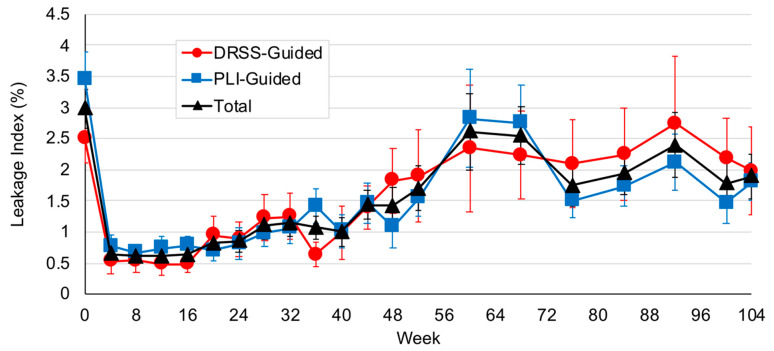
Panretinal leakage index (PLI) in through week 104. Mean PLI decreased by 11% (*p* = 0.73) and 23.6% (*p* = 0.25) compared to baseline in the diabetic retinopathy severity scale (DRSS)- and PLI-guided arms, respectively.

Through the end of the study, no significant differences were observed in change in PLI in either arm from baseline to week 104. Mean PLI decreased by 11% (*p* = 0.73) and 23.6% (*p* = 0.25) in the DRSS- and PLI-guided arms, respectively to an absolute PLI of 1.98% and 1.81% (Figure 4).

### 3.3. Indications for PLI as a Precursor to DRSS Worsening

Through week 52 of PRIME, increased PLI appeared to precede DRSS level worsening, a trend that continued through week 104. Overall, among subjects in the DRSS-guided arm who entered year 2 and achieved the initial ≥2-step DRSS improvement, 62 instances of ≥1-step DRSS worsening compared to their best DRSS were observed. 48 of these instances (77.4%) occurred simultaneously with, or were preceded by, a PLI indication for re-treatment in the visit immediately preceding the visit at which DRSS level worsening was observed. Inversely, among the same population, 57 instances of an increase in PLI above the PLI-retreatment threshold were observed; 48 of these cases (84.2%) occurred simultaneously with or were followed by a ≥1-step DRSS worsening compared to baseline in the visit immediately following the indication. Additionally, a total of 36 IAI re-treatment initiations according to DRSS were identified among this population, which occurred simultaneously with or were preceded by a PLI indication for re-treatment in 28 (77.8%) instances.

**Figure 5:**
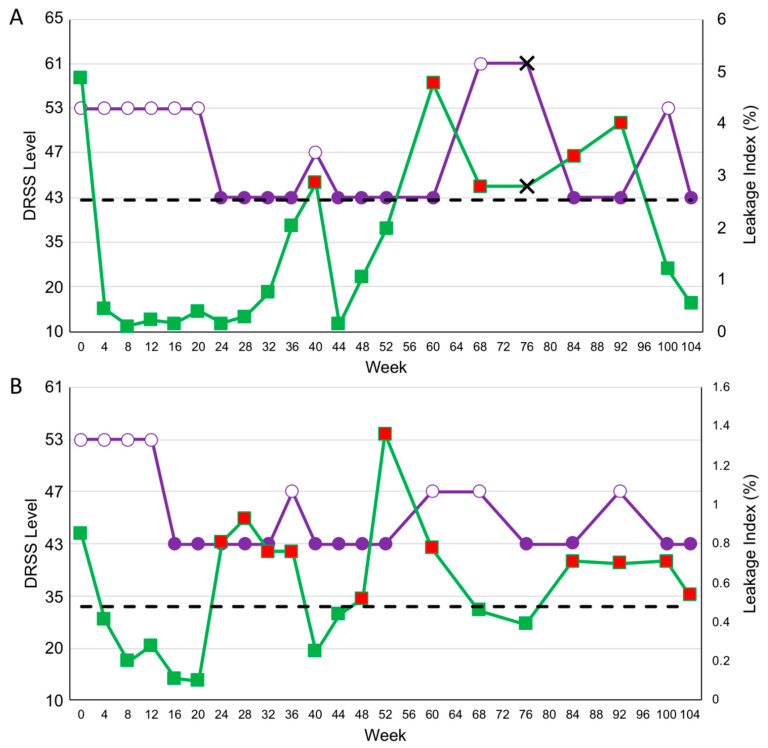
Subject cases from the diabetic retinopathy severity scale (DRSS)-guided arm through week 104. (**A**) Subject 23 and (**B**) subject 37 both demonstrated instances of ≥1-step DRSS worsening from the best-achieved DRSS, each of which was either preceded by or occurred alongside a meaningful panretinal leakage index (PLI) worsening. Purple indicates DRSS level; white-filled markers indicate a visit before the initial ≥2-step DRSS level improvement with initial monthly dosing or a visit with a DRSS worsening compared to best-achieved DRSS level; green indicates PLI level; red-filled markers indicate a worsening in PLI above the re-treatment level according to the PLI-guided protocol; black dotted lines indicate the re-treatment level as indicated by the PLI-guided protocol; black X markers indicate a missed visit.

Two specific cases in the DRSS-guided arm highlight these increases in PLI at visits preceding DRSS level worsening. The first subject (subject 23) presented at baseline with a DRSS level of 53 and PLI of 4.87% (Figure 5A). The subject had a threshold leakage of 0.16% at week 24 and if the subject had been in the PLI-guided arm, they would have been re-treated at a PLI of 2.52%. Through week 104, the subject experienced 3 visits at which a ≥1-step DRSS worsening from the best-achieved DRSS was observed, weeks 40, 68, and 100; at the visit immediately preceding the visit or at the visit at which DRSS level worsened, a meaningful PLI increase was observed. At two of these visits, those prior to weeks 68 and 100, PLI increased above their PLI-retreatment threshold.

The second subject (subject 37) presented at baseline with a DRSS level of 53 and PLI of 0.85% (Figure 5B). The subject had a threshold leakage of 0.11% at week 16 and if the subject had been in the PLI-guided arm, they would have been re-treated at a PLI of 0.48%. Through week 104, the subject experienced 4 visits at which a ≥1-step DRSS worsening from the best-achieved DRSS was observed, weeks 36, 60, 68, and 92. At the visit immediately preceding each of these visits, an increase above their PLI-retreatment level was observed.

8 of 17 PLI-guided eyes that completed the week 52 visit never experienced a ≥1-step worsening in DRSS level following the pre-defined initial ≥2-step improvement; 7 of these 8 eyes entered into year 2. Among these 7 eyes, one never experienced a ≥1-step DRSS level worsening through week 104; this eye received 6 IAI before initially achieving a ≥2-step DRSS level improvement and then received 3 PRN IAI during 13 PRN visits (20 months) through week 104. Of the remaining 6 eyes, one never regressed through W92 before becoming LTFU, after 4 IAI to ≥2-step DRSS level improvement and 5 PRN IAI during 14 PRN visits (19 months). One eye experienced DRSS worsening at week 60 after 6 months without PRN IAI, and another experienced DRSS worsening at week 68 after 14 months without PRN IAI; both of these eyes experienced an upward trend in PLI in the months preceding the DRSS worsening. The final three eyes experienced a ≥1-step worsening in DRSS level after missing at least one visit and following 7, 7, and 14 months without PRN IAI, respectively; all three eyes experienced an increase in PLI alongside DRSS level worsening.

### 3.4. Visual and OCT-Based Anatomic Outcomes through Year 2

**Figure 6:**
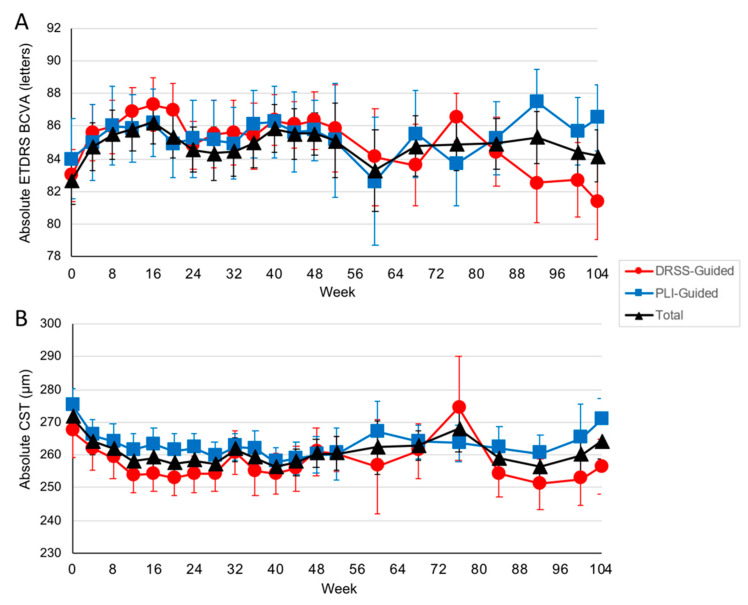
Visual and anatomic changes through week 104. (**A**) Mean absolute Early Treatment Diabetic Retinopathy Study (ETDRS) best-corrected visual acuity (BCVA) letters through week 104. (**B**) Mean absolute central subfield thickness (CST) through week 104. All error bars represent standard errors.

Through week 104, changes in visual and OCT-based anatomic outcomes were minor and similar between arms, as expected given the inclusion criteria. Among eyes that entered year 2, ETDRS BCVA increased in the DRSS- and PLI-guided arms, respectively, by 0.17 (95% CI, −2.7 to 3.4) and 0.38 (95% CI, −5.2 to 5.6) letters from baseline to week 104 to an absolute BCVA of 81.6 (approximate Snellen equivalent, 20/25; 95% CI, 82.1 to 91.0) and 86.5 (approximate Snellen equivalent, 20/20; 95% CI, 76.9 to 86.3) letters (Figure 6A); CST decreased in the DRSS- and PLI-guided arms, respectively, by −13.2 (95% CI, −39.4 to +13.0) and −5.5 (95% CI, 19.0 to +8.08) µm to an absolute CST of 254.4 (95% CI, 237.0 to 271.8) and 270.6 (95% CI, 255.9 to 285.3) µm (Figure 6B).

### 3.5. Adverse Events through Year 2

Ocular and systemic adverse events for year 2 are reported in Table 1. The incidence of DR-related adverse events was 3.4% among eyes entering year 2. No new safety signals or serious ocular adverse events occurred. There were no cases of center-involved DME development or new-onset neovascularization, and no subject received panretinal photocoagulation or vitrectomy.

Two deaths occurred in year 2 of PRIME attributed to cardiac arrest and cardiovascular disease/diabetes mellitus, respectively. Neither death was attributed to study drug or study procedure.

## 4. Discussion

The randomized PRIME trial explored the use of real-time DRSS and PLI assessments to determine re-treatment decisions for patients with DR without center-involved DME. Through week 104, all subjects in both the DRSS- and PLI-guided arms demonstrated either stable or improved DRSS compared to baseline, with all subjects requiring at least one PRN IAI during the trial, with a global mean of approximately one IAI injection required every 3.5 months.

Overall through week 104, the results of the PRIME trial were consistent with previous prospective trials reporting the need for consistent clinical follow-up and repeated anti-VEGF injections among DR patients due to the chronic, recurrent nature of the disease. While PRN trials have indicated that, once stabilized, some eyes may remain stable for long periods of time [7], cumulatively data indicate that the large majority of patients with DR will require re-treatment with anti-VEGF therapy [7,16,17], even in those with mild or apparent quiescent disease. In the current study, all patients required ongoing anti-VEGF therapy due to worsening of disease severity when treatment was discontinued. Nevertheless, in PRIME, the rate of progression of DR and the necessary re-treatment intervals were highly individualized between patients.

While overall outcomes were similar between the randomized arms in the current study, there were 3 important observations across the 2-year study.

First, in year 2, the percent of PRN visits that resulted in IAI re-treatment increased approximately two-fold in both arms. While this increase was related to the study design of reduced clinical visits in year 2, it is a clinically relevant observation. In clinical settings, patients are often transitioned to PRN regimens after a period of regular, fixed frequency dosing, and in doing so are often evaluated with less frequent visits. As demonstrated in the current dataset, among this population, when seeing patients less frequently with PRN dosing, a greater proportion of visits needing re-treatments is to be expected.

Second, a significant positive correlation was observed between the percent of PRN IAI required during year 1 and year 2. This observation is consistent with an understanding that while re-treatment intervals can vary widely among different patients receiving anti-VEGF pharmacotherapy for exudative retinal diseases, they appear to be remarkably consistent at the individual patient level. This has been elegantly demonstrated in a series of manuscripts by Fauser and colleagues in neovascular age-related macular degeneration; among this population, intravitreal VEGF suppression time after anti-VEGF treatment was intra-individually stable [18,19]. In the current series, the re-treatment intervals among patients were notably consistent longitudinally using either DRSS or PLI-guided re-treatment.

Third, PLI worsening often preceded or occurred alongside worsening in DRSS level, a phenomenon most obvious among the subjects randomized to PLI-guided re-treatment. Through week 104, 77% of instances of DRSS worsening were preceded or occurred simultaneously with a PLI indication for re-treatment, and 84% of instances of a PLI indication for re-treatment occurred alongside or before DRSS worsening, consistent with separate analyses correlating DRSS and PLI changes within patients [20]. Intuitively, this progression of disease severity aligns with current understanding of DR pathogenesis. As intraocular levels of anti-VEGF pharmacotherapy decrease in the vitreous following a bolus therapeutic injection, levels of pathologic VEGF concurrently rise, re-triggering breakdown of the blood-retina barrier leading to increased retinal vasculature permeability visualized by fluorescein leakage [21]. Subsequently, visible lesions of DR increase, including hemorrhages, leading to worsening of DR severity as observed by fundus photography [22]. Current observations suggest that PLI may prove to be a valuable biomarker for clinicians making retreatment decisions in DR patients—rising PLI levels may be indicative of a need for re-treatment with anti-VEGF therapy.

These results suggest that, at least in the context of bolus anti-VEGF monotherapy among eyes with DR without DME, regular clinical assessments and re-treatments are needed indefinitely in order to optimize outcomes. Sustained anti-VEGF therapies with next-generation approaches such as implantable devices [23], more durable pharmaceutical agents [24], and gene therapies [25,26] may be valuable to this patient population. Furthermore, treatments targeting additional mechanisms of action may also bring value to this space in the near future. For example, investigations into manipulating the Tie-2 and kallikrein systems for additive benefit among patients with DR and DME are ongoing [27,28].

Strengths of the PRIME trial include its randomized study design and its use of real-time, objective measurements of DRSS and PLI to guide re-treatment protocols. The key limitation of PRIME is the small sample size. However, given its pilot design, the results of the PRIME trial may be able to guide future, larger prospective studies evaluating biomarkers for re-treatment decisions. Another limitation is the modified version of pure DRSS grading used in the current study [14]. Additionally, there was a notably high rate of LTFU in the current study, with only 29 of 40 enrolled subjects entering year 2, and 25 subjects completing the week 104 endpoint. LTFU is a well-known challenge among patients with DR, as noted in year 1 of the PRIME trial [14] and in multiple other studies [29,30], despite the need for close clinical follow-up among this population. In the current trial, some cases of LTFU may have been due to the length of trial visits and the need for UWFA, FP, and multiple other images at each study visit. This challenge of LTFU was also exacerbated by the impact of COVID-19 during this study period. Finally, the current prospective study only considered the use of anti-VEGF management with aflibercept and did not include laser treatment options. Laser, especially pan-retinal photocoagulation (PRP), can be an excellent clinical tool either as monotherapy or in combination with anti-VEGF pharmacotherapy for the management of more advanced stages of DR.

While the 2-year results of PRIME suggest a meaningful and clinically relevant relationship between PLI and DRSS levels, with PLI increases typically preceding DRSS level worsening, the application of this to clinical practice is challenging. First, while UWFA provides remarkable insight into disease burden and severity not appreciable with fundus photography or ophthalmic examination, it is time-consuming and invasive, and therefore often not practical for all routine clinical visits; while OCT angiography (OCTA), can elegantly visualize the retinal vasculature with fast, non-invasive scan acquisition, it is not currently capable of measuring leakage index. Furthermore, PLI is not readily assessable using current commercially available technologies, and specific software systems would be required to measure and apply this biomarker in clinical practice. Lastly, the DRSS scale is not currently directly clinically applicable due to the strict methodology required to accurately grade imaging; it is our belief that advances in imaging and machine learning technology may allow for this scale to eventually be used clinically.

In conclusion, subjects enrolled in the PRIME trial were able to maintain or improve their DRSS levels through 2 years of PRN re-treatment according to real-time DRSS or PLI measurements. The lengthening of time between assessments in year 2 resulted in an increase in the proportion of visits requiring PRN IAI with subjects being treated an average of once every 3.5 months to maintain DR severity level improvements. Consistent with observations in year 1, worsening of PLI often preceded DRSS level worsening, indicating that PLI may be a valuable biomarker to guide management strategies in trials and clinical practice.

## Figures and Tables

**Table 1:** Ocular and Systemic Adverse Events in Year 2.

**Ocular Adverse Events**			
	DRSS-Guided	PLI-Guided	Total
Worsening of Cataracts	1	1	2
Worsening of PDR	1	0	1
Floaters	1	0	1
Flashes	1	0	1
Ocular Pain	1	0	1
Glaucoma	0	1	1
Cotton Wool Spots	0	1	1
**Total # of AEs**	5	3	8
**Total # of Patients**	4	3	7
**Serious Systemic Adverse Events**			
	DRSS-Guided	PLI-Guided	Total
Cerebrovascular Accident	0	1	1
Arthritic Hip Pain	0	1	1
Infection of Left Foot on Hallux	0	1	1
Bone Destruction of Right Foot	1	0	1
Pneumonia	1	1	2
COVID-19	0	1	1
Acute Chronic Renal Failure	0	1	1
Asthma	1	0	1
Worsening Anemia	1	0	1
Transient Ischemic Attack	1	0	1
Stage 2 Kidney Failure	1	0	1
Fatal Cardiovascular Disease/Diabetes	0	1	1
Fatal Cardiac Arrest	0	1	1
**Total # of SAEs**	6	8	14
**Total # of Patients**	4	6	10

DRSS = diabetic retinopathy severity scale; PLI = panretinal leakage index; PDR = proliferative diabetic retinopathy; AE = adverse event; COVID-19 = coronavirus disease 2019; SAE = serious adverse event; # = number.

## Data Availability

The data presented in this study are available on request from the corresponding author.
